# A RE-AIM Analysis of a Mental Health App for Undergraduate and Medical Students during the COVID-19 Pandemic: A Retrospective Cross-Sectional Study

**DOI:** 10.3390/ijerph20136266

**Published:** 2023-06-30

**Authors:** Krisdaniel Berreta, Cynthia Nguyen, Alexis M. Stoner, Lindsey Ridgeway, Angela Wilson, Natalie Fadel, Duke Biber

**Affiliations:** 1 Department of Preventive Medicine and Public Health, Edward Via College of Osteopathic Medicine, Spartanburg, SC 29303, USA; 2 Student Affairs, Edward Via College of Osteopathic Medicine, Spartanburg, SC 29303, USA; 3 Mayo Clinic, Rochester, MN 55905, USA; 4 Department of Psychiatry and Neuro-Behavioral Sciences, Edward Via College of Osteopathic Medicine, Spartanburg, SC 29303, USA; 5 Department of Health Promotion and Physical Education, Kennesaw State University, Kennesaw, GA 30144, USA

**Keywords:** mental health app, COVID-19, mental health, digital health

## Abstract

Objective: The purpose of this study was to use the RE-AIM framework to evaluate the implementation of a mental health app designed for undergraduate and medical students during the COVID-19 pandemic. Participants: Medical (*n* = 270) and undergraduate students (*n* = 1386) from five universities in the Appalachian region in the United States participated in this study. Methods: Universities from the United States were recruited to deploy the Sharpen app for medical and undergraduate students. The Sharpen app provided psychoeducational modules in mental health literacy, social-emotional learning, mindfulness-based stress reduction, and suicide prevention to promote protective factors for students. The utilization of the Sharpen app was analyzed using the RE-AIM framework using a retrospective, cross-sectional design. Results: Reach: A total of 12.72% of medical students and 6.00% of undergraduate students participated in the study. Efficacy: Medical students viewed significantly more pages, had a significantly higher unique page view average, and a statistically significant exit percentage when compared to undergraduate students. Adoption: A total of 100% of the universities that were recruited participated in the study. Implementation: Five out of six implementation criteria were included, indicating high implementation. Maintenance: All of the universities continued using the Sharpen app following the end of data collection, resulting in a 100% maintenance rate. Conclusions: The RE-AIM framework indicated usability and maintenance by medical and undergraduate students. Future research needs to implement a more rigorous design to determine the impact of the Sharpen app on mental health outcomes in medical and undergraduate students.

## 1. Introduction

Postsecondary student mental health is a significant health concern given the known prevalence of illness in students entering higher education and the inequity in accessing proper treatment [1]. Since the onset of the novel coronavirus disease (COVID-19) outbreak in the United States, the established concern for college and post-graduate student mental health is being increasingly explored and reviewed [2]. The rapid progression and spread of COVID-19 within the country had disturbed and displaced students from their classrooms and forced them to abruptly adapt from in-person learning to a virtual environment [3]. Education has been delivered through different online modalities prior to the pandemic; however, students were required to transition to an online learning-only environment. In a longitudinal study using smartphone and ecological assessments, it was found that in the first academic term impacted by COVID-19 college students reported increased anxiety and depression symptoms compared to prior academic terms [4]. Correlations were made with increased phone usage, decreased physical activity, and fewer locations visited with the increased amount of COVID-19-related news, which in hand noted significantly increased anxiety and depression [4]. Psychologically, students were substantially affected by the pandemic and reported increased feelings of loneliness, anxiety, and depression [5], as well as suicidal ideation [6]. In a survey of 195 students, 91% of students claimed increased levels of fear and 86% reported a reduced number of social interactions due to the virus outbreak [7].

It can be inferred that the adjustment to virtual learning created a multitude of stressors that impacted students academically, mentally, and emotionally. In the past, students had access to interventions in person; but during the pandemic, virtual and remote psychological interventions were made more accessible with mobile technologies. The rise in technology use and access to smartphones over the last decade has gained interest as a possible means of providing treatment for students. The pandemic made virtual mental health treatment a necessity, increasing the innovative use of digital mental health technology as an effective means of addressing the ongoing crisis. A meta-analysis of studies that tested the effects of smartphone interventions to manage anxiety determined that within a sample total of 1581 participants, a digital smartphone intervention had been effective and significant in reducing symptoms [8]. In addition, a study with 741 student participants found that despite low usage or no history of using a mental health app, students were overall interested in immediate access to mental health assistance [9]. Within a sample of 474 participants, one study determined that 66% of individuals believed a mobile application during the COVID-19 pandemic was significantly helpful, especially in those with a history of mental disorders [3].

Given the increasing number of college students who matriculate with a pre-existing mental health disorder or are at high risk of developing a disorder, we believe it would be helpful to explore how different student populations utilized an app during a crisis like the COVID-19 pandemic. To ensure the generalizability and appropriate dissemination of this study’s findings, we believed it was best to use the RE-AIM framework, which emphasizes the production of generalized and valid research. It has also been used to improve the implementation and translation of evidence-based findings into applicable interventions [10]. Furthermore, the RE-AIM framework has been previously utilized to evaluate mobile health (mHealth) interventions, thus making the framework appropriate for this study [11]. Given that the results of this work could subsequently inform the development of mental health interventions moving forward, the RE-AIM framework was deemed appropriate for utilization. This study used the RE-AIM framework to evaluate the implementation of a mental health app designed for college and medical students during the COVID-19 pandemic.

## 2. Materials and Methods

### 2.1. Study Design

This study utilized a retrospective, cross-sectional design.

### 2.2. Study Population

University undergraduate and medical students were recruited from five universities in the Appalachian region of the United States. Inclusion criteria were students who intentionally chose to sign up for the app and accept the Sharpen privacy terms and conditions. Participants were excluded if they were not university students at one of the respective participating schools. The total possible reach for student participation was 18,601 undergraduate students and 2123 medical students.

### 2.3. Intervention

Resiliency Technologies (RT), who is a leader in the field developed the Sharpen^®^ system to improve the shared protective factors for suicide, trauma, and mental disorders. The Sharpen app, built using the socio-ecological model, addresses these shared protective factors for mental disorders, suicide ideation, resilience, and trauma. The app contains a library of psychoeducational content to decrease stigma, improve resilience, and mental health literacy, and increase engagement with mental health treatment. Sharpen also includes a HIPAA and FERPA compliant communication network that helps providers and mission driven organizations increase outcomes in engagement and compliance with their constituents. The Sharpen system was created over the course of 15 years through interdisciplinary research collaborations and community-based documentary film projects highlighting the power of peer stories of strength and resilience. Sharpen’s peer-to-peer, documentary film content library is built using eight evidence-based protective factors shown through research to improve resiliency [12], while offering providers and patients a toolkit to assist in recovery from an array of mental disorders. The Sharpen service includes other topics that can be tailored for different populations such as financial literacy and volunteer opportunities within the community.

The Sharpen app contains a customized library of modular content that includes peer-focused documentary film footage often with individuals affiliated with the organization deploying the app with the goal of increasing engagement. The library of psychoeducational modules is grouped into four primary categories that relate directly to the shared protective factors: mental health literacy (MHL), social-emotional learning (SEL), mindfulness-based stress reduction (MBSR), and suicide prevention (SP). These four primary categories were selected because previous research supports the impact of each on resilience and overall wellness, as well as protective effects against stress, anxiety, depression, and suicidality in this population [13,14,15,16]. Example topics comprising the 500 core Sharpen MHL, SHL, and MBSR modules include childhood trauma, ACEs, toxic stress, mindfulness interventions, test anxiety, improving coping skills, healthy relationships, improving communication skills, suicide prevention, substance use prevention, disordered eating prevention, professionalism, and mental health literacy. In addition, the modules are further customized based on “user story demographics” specific to the organization deploying the app. For each of the universities that participated in this study, the Sharpen app was tailored to include mental health screening tools, community resources, organizations, and crisis response support within their specific zip code to enhance engagement.

### 2.4. Data Collection

Aggregate usage of the Sharpen platform was collected via pageviews, unique page views, and exit percentages for undergraduate and medical students. A single page view was defined as the number of times a participant visited a specific module. Unique pageviews were the number of pageviews from the same person within one session. The average time on a page was defined as the average time a user spent on a module until leaving. An exit was determined by how often visitors exited a page after visiting any number of pages on the site. Exit percentage was calculated as the number of exits/number of pageviews.

### 2.5. Procedures

Following participant recruitment, the researchers compared the aggregate engagement with the Sharpen app between undergraduate and medical students at the participating universities using the RE-AIM framework. The aggregate, deidentified usage data was collected from the Sharpen app, which was HIPAA and FERPA-compliant.

### 2.6. Data Analysis

The implementation of the Sharpen app was evaluated using the RE-AIM framework consisting of reach, effectiveness, adoption, implementation, and maintenance [10,17]. Reach refers to the proportion of individuals that participated in the study out of the total number of possible participants. This was measured as the percentage of participating undergraduate and medical students out of the total number of possible participants from the five participating universities. Effectiveness refers to the impact of the intervention on various outcomes or variability across groups and was assessed using *t*-tests to compare and determine if there were significant differences in Sharpen app usage between medical student participants and undergraduate participants. Adoption is the number of sites that participated in the study out of the total potential number of sites recruited. Implementation refers to consistency and adaptations made throughout intervention implementation. Maintenance is the number of sites that continued using the Sharpen app following the end of data collection.

## 3. Results

The Sharpen app intervention was implemented at each of the universities between August 2019 and February 2022 for a mean implementation of 1.89 years. The implementation of the Sharpen app is analyzed in the following sections using the RE-AIM framework.

### 3.1. Reach

Over the implementation of the Sharpen app, 12.72% of medical students (270 medical student participants/2123 total medical students) and 6.00% of undergraduate students (1386 undergraduate student participants/18,601 total undergraduate students) participated in the study.

### 3.2. Efficacy

Although efficacy did not include pre/post analysis or randomization, the key efficacy metrics included usage data by all participants and group comparisons of usage across the four main categories in the Sharpen app to inform future deployment and usability (see Table 1).

Overall, there was a statistically significant difference between medical students and undergraduate students in page views, unique page views, exit percentages, and usage of the four main categories. Medical students viewed significantly more pages (3.6 ± 8.2) than undergraduate students (2.5 ± 4.3), *p* = 0.0003 had a significantly higher unique page view average (2.6 ± 5.7) than undergraduate students (1.9 ± 3.3), *p* = 0.0003 and a statistically significant exit percentage (6.4% ± 0.2) when compared to undergraduate students (8.0% ± 0.2). There was no significant difference between the average time spent on a page between medical students (46.2 ± 103.9) and undergraduate students (44.7 ± 113.6).

### 3.3. Adoption

Out of a total of five possible schools that were recruited for participation, all five consented and had student participation in the intervention for a 100% adoption rate. All five of the participating universities were from the Appalachian region, including a small, private osteopathic medical school, a mid-sized public university, a small Division II athletic college, a small private college whose population includes 90% or more African American students and first-generation college students, and a large public university.

### 3.4. Implementation

The Sharpen app for each university was developed over a period of sixty days, in coordination with university administration and counselors to identify and meet key university needs. A total of 100% of the universities tailored the Sharpen app to incorporate university logos and branding, relevant campus resources for mental health support, as well as welcome videos from university-specific counselors, faculty, and students. The implementation of the Sharpen app was delivered in a consistent, asynchronous format for all participating universities and students. Given the asynchronous format without the need for a facilitator, implementation was high. In addition, 40% (2/5 universities) utilized single sign-on for students to access the Sharpen app. All of the universities paid for the Sharpen app using an annual licensing fee, of which the cost varied based on the number of students on each campus and the level of customization desired. Lastly, four of the five universities (80%) experienced changes in leadership throughout the implementation of the Sharpen intervention. 

### 3.5. Maintenance

Out of the five schools that participated in the study, all of them continued using the Sharpen app following the end of data collection, resulting in a 100% maintenance rate.

## 4. Discussion

The purpose of this study was to evaluate the utilization of a mental health app between undergraduate and medical students using the RE-AIM framework. The results of this research provided evidence that there was some difference in how medical students consume mental health content versus undergraduate students. The remainder of the discussion focuses on the evaluation of the RE-AIM framework in relation to previous research and future implications.

In terms of reach, this study utilized two of the four suggested reach criteria, which included exclusion criteria and the percentage of individuals who participated based on a valid denominator [10]. The percentage of participating individuals is consistently the most common metric of reach; however, the reach of this study (i.e., 12.72% for medical students; 6.00% for undergraduate students) was lower than previous mobile health applications [11]. The reach rate may have been lower than in former studies due to the inability to recruit participants in person due to COVID-19, the sensitive nature of the mHealth and mental health intervention, and the lack of time or overwhelm often associated with being a college and medical student. Future implementation of the Sharpen app using the RE-AIM framework could include qualitative methods to understand recruitment and representativeness of the sample to the population to enhance reach evaluations.

The efficacy of the Sharpen app was assessed via a comparison of usage rates between groups. The results indicated statistically significantly greater usage by medical students than undergraduate students. This study was the first to assess differences between types of higher education students such as medical students and undergraduate students. This pattern could suggest that medical students valued the app more and were willing to spend more time engaging with the various modules available. A systematic review of mHealth interventions utilizing more rigorous designs significantly reduced stress, depression, and substance use in adolescents and adults [18]. It is known that medical students suffer from increasing rates of anxiety, depression, and suicidal thoughts, and future evaluation of the Sharpen app should utilize an RCT design to further substantiate the current results. COVID likely exacerbated these issues, and the results of this study could imply higher Sharpen usage by medical students because of those factors [6]. These findings were valuable as they confirm that medical students, a population at high risk for mental disorders, showed interest in having a toolkit containing de-stigmatizing, psychoeducational content that can assist them during stressful times in medical school. The results also showed that college students are interested in exploring mental health literacy and resiliency topics. The efficacy evaluation was the weakest metric of the study as only the primary outcome was reported. There was no moderation analysis, attrition reported, or measure of a primary outcome in relation to a public health goal as suggested by coding recommendations for RE-AIM [10]. However, this same study found that most mHealth interventions were not evidence-based. The current study utilized previous research to develop the mHealth application with the goal of validating the app.

The adoption for this study was high because 100% of the universities where the Sharpen app was deployed participated in the study. The adoption evaluation was based on the percentage of institutions approached that participated, using a valid denominator. The adoption in this study was much higher than previous systematic review and RE-AIM evaluations of mental health and positive psychology studies, which ranged from 11% to 86% [11,19]. Adoption criteria also included characteristics of participating institutions; however, this was not conducted in comparison to non-participating institutions as 100% of recruited universities agreed to participate. That said, other key adoption criteria were not included, such as setting exclusions, and use of qualitative data collection to assess adoption decisions, each of which could enhance future evaluation [10].

Implementation of this study was high. The Sharpen app delivered asynchronous content, allowing for fidelity to the intervention. The cost of the Sharpen app was scaled based on university enrollment, and the process of enrollment differed by university including traditional sign-on and single sign-on using university credentials. Lastly, input was elicited from key leadership at each university to help determine the best methods of implementation and content implemented. Based on RE-AIM dimensions and criteria outlined by Gaglio and colleagues (2013) [10], five of the six criteria were utilized in this study. Furthermore, input from the university leaders and staff guided the development and deployment of the Sharpen content, which is considered best practice for programming [20]. The implementation was a strength in this study as previous mHealth RE-AIM studies exhibited lower implementation and issues when reporting implementation fidelity [11]. Previous research highlights the importance of assessing program implementation with as many of the aforementioned criteria to determine true process and outcome effectiveness [21,22].

Maintenance for this study was also high as 100% of the universities that deployed the Sharpen app continued implementation following the end of data collection. Maintenance is often underreported in evaluations of programs [23]. This current study reported maintenance and also had a high maintenance rate, which is another strength when compared to previous research [11,24]. In terms of continued evaluation, it is necessary to utilize mixed methods to accurately assess maintenance and determine interrelated factors that impact the success of programming.

### 4.1. Study Limitations

One limitation of this study was the smaller sample size of medical students compared to college students. Given the larger number of college student data accessed in this study, there may be a greater representation of the population than that of medical students. This could be addressed in future research by incorporating a larger medical student population and utilizing an app across multiple school campuses or extending it to other nearby medical schools in the Appalachian region. A second potential limitation was that the app was advertised as a resource for mental health and as such, the data may have been skewed by students who already had an established interest in seeking out mental health resources. Baseline knowledge and interest in mental health was not measured in this study, which would give more specific information on the needs and interests of these groups with regard to mental health technology. Lastly, this study did not utilize a comparison group with randomization, nor pre- and follow-up testing of efficacy. This study simply analyzed efficacy through usability, and future research should include more rigorous analysis.

### 4.2. Future Research Implications

As past research and the present study suggests, there is an interest amongst both undergraduate and medical students in a digital mental health resource such as an app [9]. Our findings would be useful for the development of a targeted mental health app that would contain multi-dimensional resources for primary, secondary, and tertiary mental health prevention. Increasing the usability of a mental health app for these populations could bridge the access to care for this age group. There is a need for research that addresses the ongoing issue of poor mental health amongst the undergraduate and graduate student population; we suggest that future researchers take advantage of these findings when implementing resources. It would also be useful to study more specifically individual usage over time and measure the impact on mental health. Lastly, the inclusion of all sub-dimensions of the RE-AIM framework using a rigorous, randomized controlled trial would greatly enhance the evaluation and translation of the Sharpen app.

## 5. Conclusions

From our study, it is evident that medical students and undergraduate students appear to have an interest in topics that improve protective factors for mental health. A larger sample size for future studies can help mental health app providers better disseminate targeted content to various student populations. Our study identified MHL, MBSR, and SEL as areas of interest for students during the onset of the COVID-19 pandemic. This study offers insight for further investigation into the dissemination and implementation of mental health technology solutions that can help address the mental health crisis.

## Figures and Tables

**Table 1:** Comparing Sharpen usage between undergraduate and medical students.

	Medical		Undergraduate		Sig.
Mental Health Literacy Medical (N = 253) Undergraduate (N = 332)	M	SD	M	SD	*p*-value
Page Views	3.61	6.33	2.92	4.19	>|t| = 0.1322 >t = 0.0661 <t = 0.9339
Unique Page Views	2.76	4.14	2.26	2.99	>|t| = 0.1027 >t = 0.0513 <t = 0.9487
Exit Percentages	0.07	0.18	0.10	0.24	>|t| = 0.0956 >t = 0.09522 <t = 0.0478 *
Social-Emotional Learning Medical N = 261 Undergraduate N = 487	M	SD	M	SD	*p*-value
Page Views	2.57	2.93	1.95	1.76	>|t| = 0.0020 * >t = 0.0010 * <t = 0.9990
Unique Page Views	1.93	1.75	1.46	1.10	>|t| = 0.0001 * >t = <0.0001 * <t = 0.9999
Exit Percentages	0.05	0.18	0.07	0.22	>|t| = 0.1037 >t = 0.9482 <t = 0.0518
Mindfulness-Based Stress Reduction Medical N = 233 Undergraduate N = 406	M	SD	M	SD	*p*-value
Page Views	2.71	3.12	2.11	3.47	>|t| = 0.0251 * >t = 0.0125 * <t = 0.9875
Unique Page Views	2.09	2.14	1.67	2.39	>|t| = 0.0232 * >t = 0.0116 * <t = 0.9884
Exit Percentages	0.10	0.26	0.06	0.21	>|t| = 0.0305 * >t = 0.0153 * <t = 0.9847
Suicide Prevention Medical N = 21 Undergraduate N = 73	M	SD	M	SD	*p*-value
Page Views	1.24	0.54	1.71	2.16	>|t| = 0.0920 >t = 0.9540 <t = 0.0460 *
Unique Page Views	1.24	0.54	1.40	1.30	>|t| = 0.4101 >t = 0.7950 <t = 0.2050
Exit Percentages	0.02	0.11	0.10	0.28	>|t| = 0.0554 >t = 0.9723 <t = 0.0277 *

* Indicates significant *p* value ≥ 0.05.

## Data Availability

No new data were created or analyzed in this study. Data sharing is not applicable to this article.
